# Photoelastic and Finite Element Analyses of Occlusal Loads in Mandibular Body

**DOI:** 10.1155/2014/174028

**Published:** 2014-10-08

**Authors:** Ana Cláudia Rossi, Alexandre Rodrigues Freire, Felippe Bevilacqua Prado, Luciana Asprino, Lourenço Correr-Sobrinho, Paulo Henrique Ferreira Caria

**Affiliations:** ^1^Piracicaba Dental School, State University of Campinas, UNICAMP, Piracicaba, SP, Brazil; ^2^Department of Morphology, Anatomy Area, Piracicaba Dental School, UNICAMP, Limeira Avenue 901, P.O. Box 52, 13414903 Piracicaba, SP, Brazil

## Abstract

This study proposed to evaluate the mandibular biomechanics in the posterior dentition based on experimental and computational analyses. The analyses were performed on a model of human mandible, which was modeled by epoxy resin for photoelastic analysis and by computer-aided design for finite element analysis. To standardize the evaluation, specific areas were determined at the lateral surface of mandibular body. The photoelastic analysis was configured through a vertical load on the first upper molar and fixed support at the ramus of mandible. The same configuration was used in the computer simulation. Force magnitudes of 50, 100, 150, and 200 N were applied to evaluate the bone stress. The stress results presented similar distribution in both analyses, with the more intense stress being at retromolar area and oblique line and alveolar process at molar level. This study presented the similarity of results in the experimental and computational analyses and, thus, showed the high importance of morphology biomechanical characterization at posterior dentition.

## 1. Introduction

The form and function of human mandible report that the region of the mandibular body reshapes itself forward of the stresses generated in the teeth and muscle action [1, 2]. Functionally, teeth and masticatory muscle stimulate and activate the formation and organization of the mandibular bone tissue [3].

To verify the behavior of the human jaw, depicting the efforts of chewing allows determining the stress distribution and recognizing areas with greater bone strength [2]. The mandibular biomechanics evaluate the responses of bone under mechanical stimuli and the characteristics of the distribution stress. Also, the knowledge of stress distribution in mandible contributes to understand the effect of surgical approaches (e.g., orthognatic surgery) and action of occlusal forces on implant-supported prostheses and, thus, to reach the minimal damage to the bone support structures. [2, 4]. In addition, anatomical characteristics in presence of mechanical stimulation and muscular action influence the form of mandibular bone [5].

The literature reports that structures such as the oblique line, body, and base of the mandible concentrate stress from masticatory loads [6]. However, according to Schwartz [2], this concentration pattern, due to mandible morphological characteristics, may generate different results according to the method of simulation used for each case. In addition, more knowledge is needed based on the mechanisms of remodeling and how loads are acting on the mechanical environment of the masticatory system [7].

Our hypothesis was that the mandibular body is designed to resist the occlusal loads of mastication with respect to experimental investigations which is contingent on certain inferences, while it may be that, within restricted regions of the mandibular corpus, occlusal loads produce relatively large stress distribution as alveolar process.

Thus, the aim of the study was to characterize, in mechanical condition, this region through the stress distribution based in photoelastic analysis and finite element analysis (FEA). FEA was performed to determine the importance of mandibular geometry in posterior region of body under occlusal load, through the energy dissipation caused by the routine masticatory loads on the posterior region.

## 2. Materials and Methods

This study was approved by the Committee for Ethics of Research of the State University of Campinas (protocol 005/2011).

**Figure 1:**
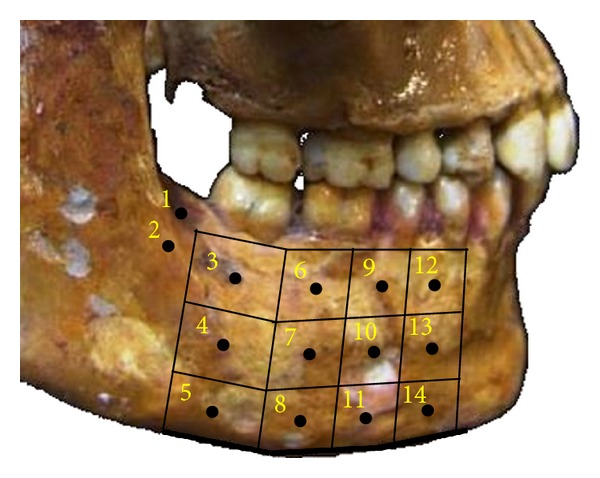
Schematic representation of the division and numbering of the areas evaluated in the mandibular body.

First, for two analyses, the area of the mandibular body used in this work was divided into 14 areas to facilitate the analysis of the stresses (Figure 1). These areas were numbered as [6] 1 (retromolar trigon), 2 (oblique line), 3 (alveolar process at level of the of 2nd molar), 4 (midbody at level of the 2nd molar), 5 (mandibular base at level of the 2nd molar), 6 (alveolar process at level of the 1st molar), 7 (midbody at level of the 1st molar), 8 (mandibular base at level of the 1st molar), 9 (alveolar process at level of the 2nd premolar), 10 (midbody at level of the 2nd premolar), 11 (mandibular base at level of the 2nd premolar), 12 (alveolar process at level of the 1st premolar), 13 (midbody at level of the 1st premolar), and 14 (mandibular base at level of the 1st premolar).

### 2.1. Photoelastic Analysis

Photoelastic resin model (Araldite epoxy resin, Araltec Chemicals Products Ltda, Huntsman) of adult dentate macerated hemimandible and of the respective antagonist posterior teeth were obtained by replication of the natural mandible. The antagonist teeth were duplicated to simulate the occlusion with the lower posterior teeth.

**Figure 2:**
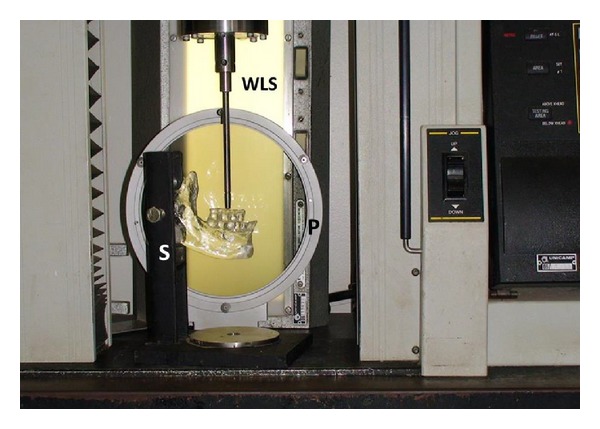
Application of the load in the photoelastic analysis. WLS: white light source; P: polarizing filter; S: support.

Load tests were performed in an Instron Model 4411 (Instron Corp, Norwood, MA) universal testing machine equipped with polariscope (white light source and polarizing filter), and digital camera (Sony Model Handycam DCR-SR300 6.1 MP, Sony Corporation, Japan). Photoelastic resin model was placed in a support set by the equipment load testing (Figure 2).

For the load test, we applied consecutive vertical loads with 50, 100, 150, and 200 Newtons (N) at a fixed point of the axis of upper 1st molar [9] (Figure 2).

In general, photoelastic analysis demonstrates the quality, location, and distribution of stresses in an object by fringe patterns that appear as a series of successive and contiguous different-colored bands (isochromatics) in which each band represents a different degree of birefringence corresponding to the underlying stress in the tested part. The contour of an isochromatic fringe is determined by the distribution of stresses in that particular region and represents equal differences in principal stresses [10].

In this study, to evaluate the stress generated on the lateral face of the jaw, images showing isochromatic fringes of each load application were obtained using a digital camera equipped with an optical filter lens. During each loading sequence, isochromatic fringes in the resin were observed and photographed, white light polarized optical effects are manifested as colored fringes which have a fringe order according to the load intensity. The fringe order at a certain point is related to the level of stresses in the model. Closer to the red color areas means higher stress concentration. Closer to the white color areas means lower stress concentration [4].

We performed the qualitative analysis of the fringes. The number and order of the fringes indicate stress intensity, whereas the proximity between them represents stresses concentration. The stress distribution is observed through the isochromatic fringes, and each fringe order is counted by the transition of colors [11]:

- fringe of order = 0 (transition white/black),
- fringe of order = 1 (transition red/blue),
- fringe of order = 2 (transition red/green),
- fringe of order = 3 (transition pink/green).

**Figure 3:**
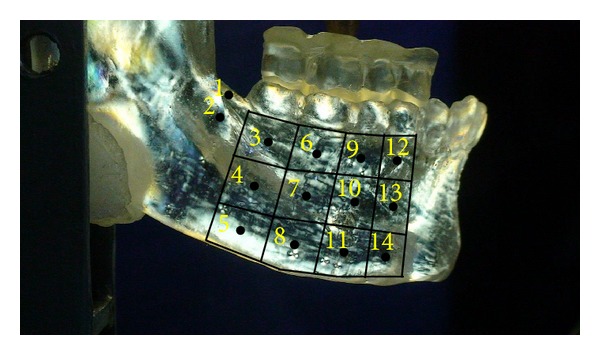
Schematic representation of the division and numbering of the 14 areas evaluated in the mandibular body on the epoxy resin model.

This qualitative analysis of the fringes was performed in each of the 14 areas (Figure 3).

### 2.2. FEA

The CT (GE HiSpeed NX/i CT scanner, General Electric, Denver, CO, USA) was performed at the same hemimandible used to perform the replicas described above. To increase the accuracy in the geometry, the CT slices had 0.25 mm of thickness.

**Figure 4:**
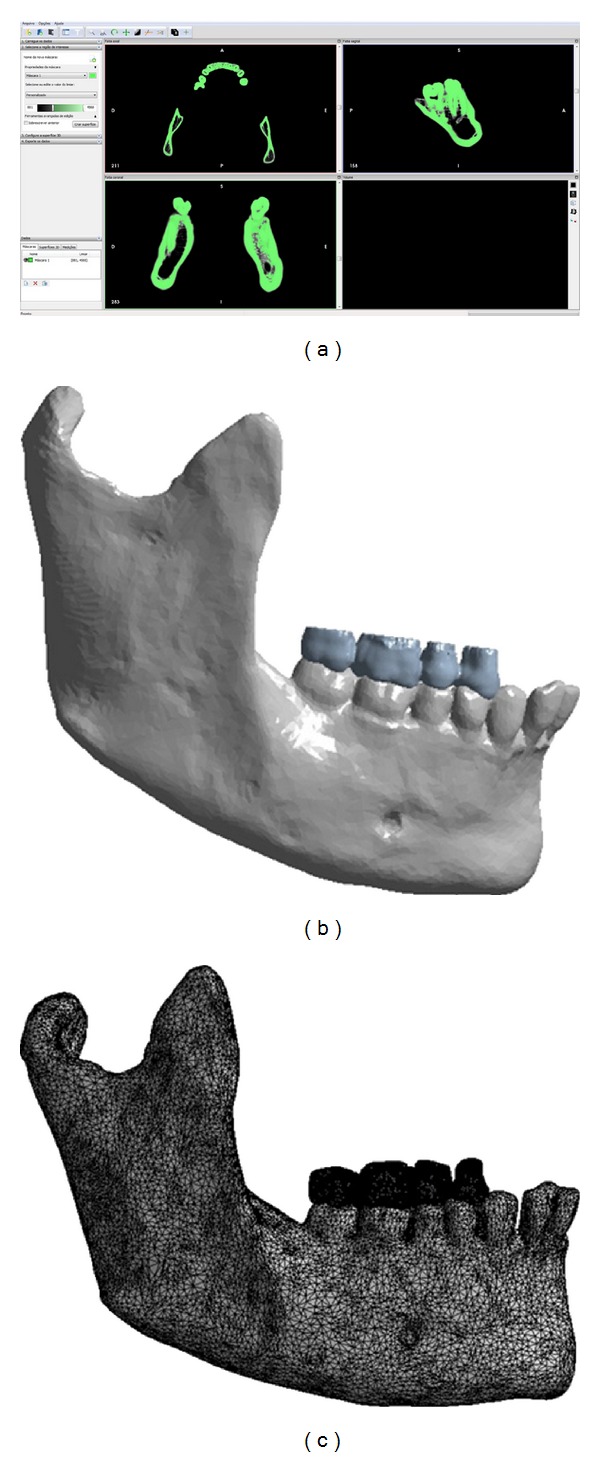
(a) Selection of bone and teeth in software InVesalius 3.0b on CT. (b) CAD model. (c) Finite element model (FE mesh).

The bone structure and teeth were selected according to the color of pixels, using threshold values in units HU (Hounsfield Units) using InVesalius 3.0b software (Center for Information Technology “Renato Archer,” São Paulo, Brazil) (Figure 4(a)).

The structures were converted in a three-dimensional model with stereolithographic file format (STL), which was the basis for modeling the geometry CAD (computer-aided design) (Figure 4(b)) for FEA [12]. For this purpose, we used the software Rhinoceros 5.0 (McNeel & Assoc., USA).

The FEA was performed in the software Ansys v14 (Canonsburg, PA, USA). The geometries (CAD model) of the hemimandible and antagonists teeth were imported to the software Ansys v14 for construction of the finite element mesh (Figure 4(c)) in which the split occurred on the solids surface. The finite element mesh presented tetrahedral elements with 208388 elements and 352097 nodes.

The anatomical structures were characterized as the mechanical properties of photoelastic resin (epoxy resin) and bone (Table 1), both as isotropic structures.

Differences of mechanical properties in the model may influence the results [13–15]. Since the aim of the study involves structural responses related to morphology, numerical values were applied to bone structure and also involved teeth. This concept has been determined from experimental studies applied to the FEA in human skulls, which determined that only the geometry was sufficient for understanding the biomechanics craniofacial [16]. Previous studies suggests that neither general stress-strain patterns nor mastication loads are greatly affected by varying material properties [8, 13, 15, 17]; however, increased precision regarding properties will lead to more accurate predictions of actual stress-strain magnitudes.

To determine the regions of high and low stress compared with the photoelastic analysis, the stress values were obtained excluding the direct relationship with the biological response. The mechanical properties of the resin epoxy were considered in order to reproduce the same situation of the mechanical testing in the computer simulation [18]. Thus, we could assess the reliability of the analysis settings (boundary conditions and loading) in the computational simulation related to the experimental analysis.

**Figure 5:**
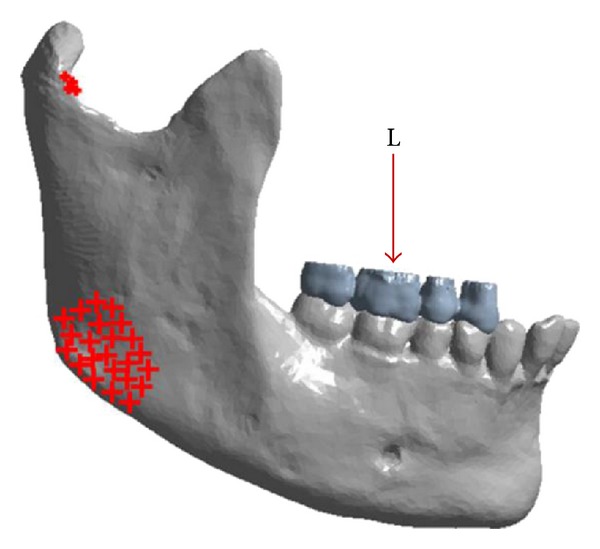
Representation of the restriction system (insertion of the masseter, medial pterygoid, and lateral pterygoid muscles) and load (L) applied to the computational model in Ansys v14 software.

The restrictions were applied in the region of the insertion of the masseter and medial and lateral pterygoids preventing free movements in the axes *x*, *y*, and *z* during the simulation. Loads with 50, 100, 150, and 200 N [9] were applied at a fixed point of the axis of the upper 1st molar (Figure 5).

Results were evaluated according to the equivalent von Mises stress distribution. The von Mises stress is a representation of the effective stress which are caused by energy flow along the material whose is receiving a load. Their magnitudes reflect the mechanical behavior of the structure and can be represented numerically or as a color-coded projection onto the model geometry.

**Figure 6:**
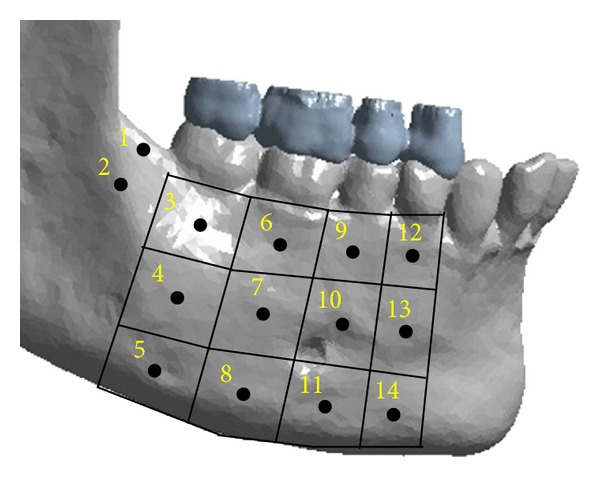
Schematic representation of the division and numbering of the 14 areas evaluated in the mandibular body on the computational model.

Stress values of von Mises were obtained at each point, three times by the same examiner, in the assessed areas (Figure 6) to facilitate the evaluation and to confirm the correct region selection for analysis. For this purpose, the reproducibility of the evaluations was obtained by the intraclass correlation coefficient (ICC) in BioEstat 5.0 software (Mamiramuá Foundation, Pará, Brazil).

## 3. Results

### 3.1. Photoelastic Analysis

The orders of patterns of isochromatic fringes generated in each region are shown in Table 2.

**Figure 7:**
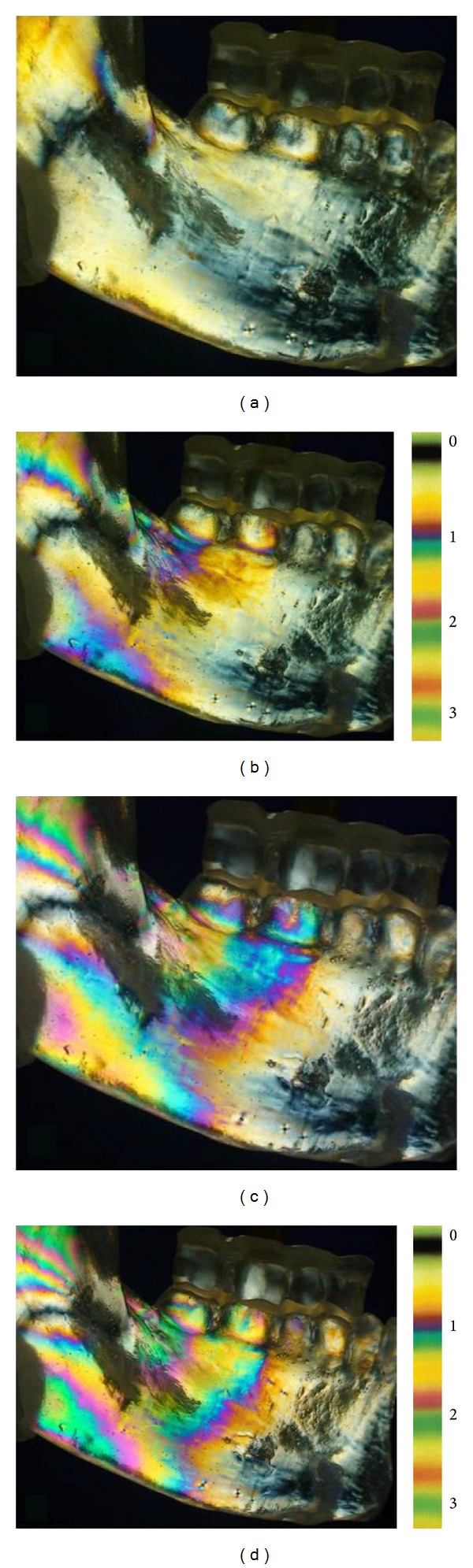
Distribution of isochromatic fringes in photoelastic analysis. Right, scale of the orders of isochromatic fringes. (a) 50 N, (b) 100 N (c) 150 N, and (d) 200 N.

Under the initial load of 50 N, the stresses were concentrated only in the 1 and 5 areas with order fringes of 1 (Figures 6 and 7(a)). Under 100 N load, the mandibular stresses were concentrated in the 1, 2, 3, 4, 5, and 6 areas with order fringes of 1 (Figures 6 and 7(b)).

When loads with 150 N were applied, the stresses were concentrated in 1, 2, 3, and 4 with order fringe of 2, and in the 5, 6, 7, and 8 areas with order fringe of 1 (Figures 6 and 7(c)). Loads with 200 N resulted in stresses concentrated in the 1, 2, 3, and 4 areas with order fringes of the 3; 5 and 6 areas present order fringes of 2; 7, 8, 9, 10, and 11 present order fringes of 1 (Figures 6 and 7(d)).

In all loads applied there was no stress concentration in the 12, 13, and 14 areas.

### 3.2. FEA

In either mechanical property applied in the finite element models, epoxy resin, or bone, for all loads and in all evaluated areas, the reproducibility was excellent 1.000 (*P* < 0.0001).

**Figure 8:**
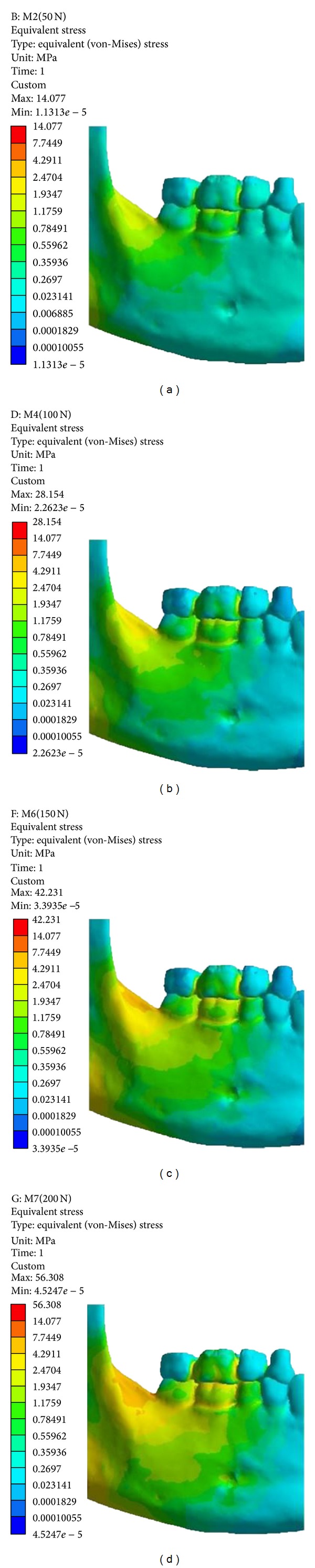
Von Mises stresses of model characterized as epoxy resin. (a) 50 N; (b) 100 N; (c) 150 N; (d) 200 N.

**Figure 9:**
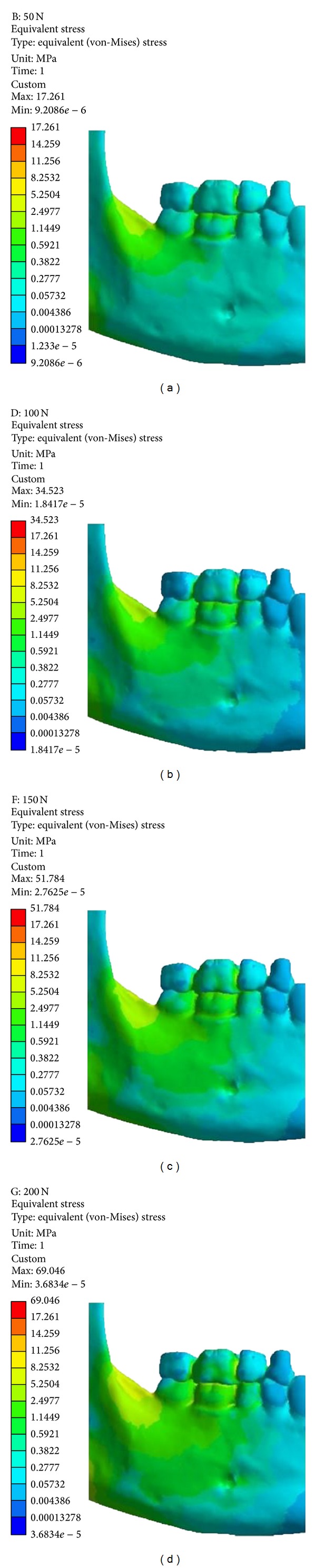
Von Mises stresses of model characterized as bone. (a) 50 N; (b) 100 N; (c) 150 N; (d) 200 N.

The von Mises stresses were distributed similarly in both the characterized models, epoxy resin (Figure 8), and bone (Figure 9). Higher intensities of stresses occurred in the molars regions, that is, 1, 2, 3, and 6 (Table 2).

In the model characterized as bone, the stresses showed lower values (Table 2) due to the physical properties of the bone to present more rigid properties (values) than the epoxy resin.

In all applied loads and in both models, the values of the von Mises stresses were less intense in 12, 13, and 14 areas (Figures 7 and 8).

## 4. Discussion

Under polarized light, the distribution of internal loads in transparent materials shows different color patterns that are studied by photoelastic analysis [4]. In comparison, FEA is a numerical tool to assess stress data, such as deflection and displacement, in computationally elaborate models. FEA is considered an effective biomechanical method to evaluate complex geometries, as bony structures of vertebrate [19]. Here, we found that both analyses were useful to evaluate the functional mechanical response of structures upon masticatory effort, based on the material properties.

We verified that the mechanical response occurred initially in areas with major bone strength, thickness, and density mainly in the oblique line and mandibular base, in agreement with the data shown by Schwartz-Dabney and Dechow [6]. The oblique line and retromolar trigon are important structures in the distribution of stresses from mastication [1]. Sicher and DuBrul [20] previously suggested that the midbody and the mandibular base could have a structural organization that receives the stresses. This hypothesis, although without experimental basis, reforced the concept of ideal mechanical environment of deformation which maintains the morphology by mechanical function [21].

Specifically, in the lower molars region, the concept of morphological adaptation through mechanical function is supported by morphological questions (mandible form in this region) [22] and functional adaptation (relationship between the presence and absence of teeth with biological response) [7]. There is a controversy regarding the occlusal loads which lead to variations in the stresses between the alveolar process and mandibular base. Studies using photoelastic analysis and FEA suggest that occlusal loads concentrate their stresses in the alveolar process [1, 23–26].

Although the alveolar process is a structure of lesser thickness than the midbody and the mandibular base [6], the results of this study showed greater intensity in the alveolar process (molar region) (fringes of order 2 in photoelastic analysis and von Mises stresses with values of 2.23 MPa and 1.51 MPa at the second and first molar, resp.). This result reinforces the concept that the alveolar process is an anatomical structure adapted to receive occlusal loads of major magnitude. This adaptation is associated with the concept that areas of major strength with minimum presence of materials are present in the bony structures [21]. In addition, Roberts et al. [27] showed that alveolar process is the denser area of the cortical bone, resulting in greater absorption of loads.

Greater stress concentration was observed in periodontal and peri-implant regions, using both photoelastic analysis [10, 28] and FEA [16]. These studies used simple geometries (in blocks) for bone support reproduction. Here, we used geometry similar to the physical structure. Thus, it can be noted that the molar alveolar process is with major stress demonstrating the importance of maintaining the bone during mastication in this region and helps to understand the presence of severe resorption in the absence of posterior teeth and the high rate of failures of implants placed in the mandibular body [28].

A study using photoelastic analysis showed that, under the action of mastication muscles, the mandibular ramus suffers high stress affecting the mandibular body [29]. In addition, bone structure in regions 1, 2, 3, 4, 5, 7, and 8 presented higher density and thickness compared with the other assessed regions [6]. We verify, in both analyses, that the action of the posterior occlusal loads promotes elevated stress concentration especially in the regions described above. Thus, these data support the concepts of mechanical adaptation and morphological changes due to loss of function in this region [7, 22]. Experimental and computational analysis in biomechanical studies shows that bones geometries have major sensibility to mechanical stimuli since the morphology is an important factor in this type of response [14]. We observed that the regions of greatest stress concentration were common in both analyses. This relationship was demonstrated by the similarity between the stress distribution in the FEA with model characterized by resin and model characterized as bone. However, the differences between the values were not considered for the comparison among the analyses since the model was simplified in relation to the different materials present in the human mandible (compact bone, cancellous bone, and dental structures). The application of FEA to study the relationship morphology/biomechanics is effective, as demonstrated by other authors [8].

The limitation of our study regarding the bone material property values is the isotropic properties applied to the bone geometry, in which the material is linear, elastic and homogenous. These features are different to the bone anisotropy and non-homogeneous features, as occurs in living tissue [14]. In addition, we used a hemimandible due to the limitation of photoelastic analysis, in which it is necessary to allow the passage of the light. As the photoelastic analysis was important to validate the reproduction of computational geometry of mandible, such characteristics were kept in the simulation. In this case, the construction of hemimandible geometry resulted in the anatomical fidelity in experimental and computational analyses. These model characteristics can determine the importance of the geometry, which is fundamental to mechanical performance, in routine masticatory loads simulation [15]. Moreover, the loads applied in this study were not dynamic, but static, although in previous studies, the stress distribution and magnitude by static analysis were almost consistent with those by dynamic analysis [1, 3, 4].

## 5. Conclusions

1. In both analyses, occlusal loads of 100, 150, and 200 N generated stresses concentrated in the 1, 2, 3, and 4 areas.
2. Both analyses showed that the mandibular body concentrates stresses in the retromolar area, oblique line. and alveolar process at the level of the molars when submitted to stresses in the first molar.
3. In comparison of an FEA model with the Epoxy resin or bone properties, although the material properties have been different, the disposition of areas of stress concentration was similar, reinforcing the geometry factor for determining the results.

## Figures and Tables

**Table 1:** Mechanical properties of the anatomical structures.

Material	Young's modulus (MPa)	Poisson's ratio
Resin*	3102,6	0,30
Bone [8]	14000	0,30

*Araltec Chemicals Products Ltda, Hunstman, MPa = megapascal.

**Table 2:** Isochromatic fringes order values and von Mises stress (in MPa) to computational model characterized as resin and as bone.

Area	Isochromatic fringes order				Von Mises stresses (epoxy resin)				Von Mises stresses (bone)			
	50 N	100 N	150 N	200 N	50 N	100 N	150 N	200 N	50 N	100 N	150 N	200 N
1	0	1	2	3	1,64	3,65	5,35	6,89	1,77	3,93	5,00	6,59
2	1	1	2	3	0,89	1,86	2,60	3,75	0,85	1,91	2,17	3,03
3	0	1	2	3	0,76	1,39	2,36	3,06	0,58	1,13	1,73	2,23
4	0	1	2	3	0,46	0,83	1,26	1,83	0,37	0,69	0,97	1,41
5	1	1	1	2	0,20	0,48	0,71	2,03	0,11	0,28	0,40	0,54
6	0	1	1	2	0,48	1,03	1,49	2,03	0,39	0,74	1,11	1,51
7	0	0	1	1	0,29	0,71	1,06	1,45	0,25	0,46	0,74	0,92
8	0	1	1	1	0,18	0,34	0,53	0,80	0,14	0,20	0,36	0,49
9	0	0	0	1	0,20	0,41	0,68	0,90	0,14	0,26	0,43	0,90
10	0	0	0	1	0,15	0,31	0,72	0,93	0,12	0,20	0,36	0,45
11	0	0	0	1	0,12	0,2	0,32	0,40	0,11	0,14	0,2	0,33
12	0	0	0	0	0,11	0,24	0,28	0,38	0,07	0,16	0,24	0,32
13	0	0	0	0	0,09	0,21	0,33	0,41	0,07	0,12	0,22	0,28
14	0	0	0	0	0,06	0,13	0,23	0,34	0,04	0,07	0,13	0,15
